# Sub-50-femtosecond gain-managed amplified pulses enhance nonlinear ablation efficiency

**DOI:** 10.1364/BOE.582102

**Published:** 2025-12-10

**Authors:** Liam J. Price, Kai Zhang, Nicholas J. Otero, Paul Repgen, Pablo A. Valdes, Frank Wise, Fatih Ömer Ilday, Bryan Q. Spring

**Affiliations:** 1 Translational Biophotonics Cluster, Northeastern University, Boston, Massachusetts 02115, USA; 2 Department of Physics, Northeastern University, Boston, Massachusetts 02115, USA; 3 Department of Physics and Astronomy, Ruhr University Bochum, 44801 Bochum Süd, Germany; 4 Department of Electrical Engineering and Information Technology, Ruhr University Bochum, Bochum Süd 44801, Germany; 5 Department of Neurosurgery, University of Texas Medical Branch, Galveston, Texas 77555, USA; 6 Department of Applied and Engineering Physics, Cornell University, Ithaca, New York 14850, USA; 7 Department of Bioengineering, Northeastern University, Boston, Massachusetts 02115, USA

## Abstract

Nonlinear femtosecond (fs) laser ablation enables highly localized energy deposition for cell microsurgery. Conventional systems operate at either low (∼1 kHz, amplified µJ pulse energy) or high (∼80 MHz, unamplified low nJ) repetition rates, but intermediate rates with amplified pulse energy offer a promising balance of ablation speed and thermal control. We custom-built a low-cost, 32 MHz femtosecond fiber laser system with gain-managed nonlinear amplification, boosting pulse energy from 5 to 90 nJ while compressing pulse duration to 46 fs. In this intermediate–repetition-rate regime, the use of sub-50-fs pulses enhances ablation efficiency by strengthening multiphoton absorption and lowering the effective ablation threshold, while also leveraging multi-pulse incubation effects that promote cumulative energy deposition at reduced per-pulse energies. Compared to 200–500 fs pulses typically used for ablation, shorter durations double ablation efficiency in silicon and yield a ∼10× increase in cell membrane damage. In 3D tumor models, this approach enables targeted subsurface ablation up to 400 µm depth with 6 nJ pulse energy. These results inform the design of next-generation femtosecond laser systems for microsurgery.

## 1. Introduction

Nonlinear femtosecond laser ablation shares several advantages with multiphoton imaging, including intrinsic spatial confinement of energy deposition. This localization limits off-target effects and enables precise, three-dimensional interaction with biological tissues. When laser wavelengths are selected to avoid strong linear absorption, efficient power delivery to deep focal volumes becomes possible. Femtosecond pulses have been shown to remove material with submicron precision through nonlinear interactions [1–7], enabling a wide range of materials processing and cell nanosurgery applications. Although clinical implementation is beyond the scope of this work, here we provide a detailed characterization of nonlinear laser ablation under previously underexplored conditions, with the aim of informing future development of precise, biologically compatible ablation technologies.

Femtosecond laser ablation is typically categorized into two operational regimes based on repetition rate. At low repetition rates ( ≤ 1 MHz), pulse energies range from hundreds of nanojoules to several microjoules, often sufficient to drive single-pulse ablation through plasma-mediated breakdown [1,2,6,8–14]. In contrast, high repetition rate systems ( ≥ 100 MHz) frequently use unamplified oscillators producing pulses of 10s of nanojoules or less, relying on cumulative effects from multiple pulses, such as free-electron mediated decomposition, multiphoton-based photochemical damage, or thermal accumulation, to achieve ablation [5,15–24].

Each regime has advantages and limitations. Low-repetition-rate systems allow high-energy, single-pulse ablation but risk excessive collateral damage, or reduced efficiency from optical shielding, particularly when using long pulse durations [16,25]. High-repetition-rate systems offer smoother energy deposition but are constrained by low pulse energies potential overheating due to high average powers and even multi-pulse optical shielding if the laser scan speed is insufficiently fast [17]. These tradeoffs motivate interest in an intermediate repetition rate regime, which may combine high pulse energies with high pulse counts, potentially enhancing ablation throughput without significantly sacrificing precision. An emerging alternative, termed the ablation-cooling regime, utilizes GHz intraburst pulse trains combined with kHz–MHz interburst repetition rates to ablate material faster than heat can diffuse, thereby enabling efficient ablation with intrinsic thermal confinement [26].

For low- and high-repetition-rate regimes, the majority of femtosecond ablation studies have used pulse widths of ∼ 100 fs to several picoseconds [1,2,9–13,27]. Although several reports have examined shorter pulse durations [6,15,27], and others have characterized the impact of pulse width on ablation efficiency in specific materials [28–32], these investigations have largely been conducted within the traditional ablation regimes—either low repetition rate, where ablation is dominated by single-pulse plasma formation, or high repetition rate, where thermal accumulation and reactive oxygen species (ROS) generation become significant. In contrast, the present study examines how further reducing pulse duration influences ablation efficiency in the intermediate-repetition-rate regime, in which material removal results from the accumulation of many femtosecond pulses at lower average powers than those typical of high-repetition-rate systems with comparable pulse energies.

To this end, we constructed a custom femtosecond fiber laser system operating at 32 MHz, positioned between conventional low- and high-repetition-rate regimes. A compact, low-cost all-normal-dispersion (ANDi) oscillator produces 70 fs pulses with 5 nJ per pulse [33]. To scale up pulse energy while further compressing pulse duration, we integrated a gain-managed nonlinear amplifier (GMA) [34–36], which increased output pulse energy to 90 nJ and shortened pulse duration to 45 fs. Unlike traditional chirped-pulse amplification, GMA supports high-energy amplification that yields generally shorter pulse durations with a simpler system integration [34]. This capability makes GMA well suited for bio-ablation applications where compactness, tunability, and repetition rate scalability are essential.

In this work, we use the resulting system to investigate how pulse duration affects nonlinear ablation efficiency in both silicon and biological cells. The strong dependence of multiphoton absorption on temporal photon density suggests that shorter pulse durations should enhance ablation yield. While this dependence is acknowledged in the context of imaging and general ablation theory [37,38], it has not been systematically quantified in the transitional regime between ∼ 1 MHz and ∼ 100 MHz. Our data show that, at constant pulse energy, reducing pulse duration from ∼ 200 fs to ∼ 40 fs yields significant improvements in ablation efficiency across both inorganic and biological targets. This direct investigation of pulse-duration effects at intermediate repetition rates bridges an important knowledge gap and supports the optimization of nonlinear laser ablation systems towards high-speed microsurgical applications.

## 2. Methods

**Fig. 1.**
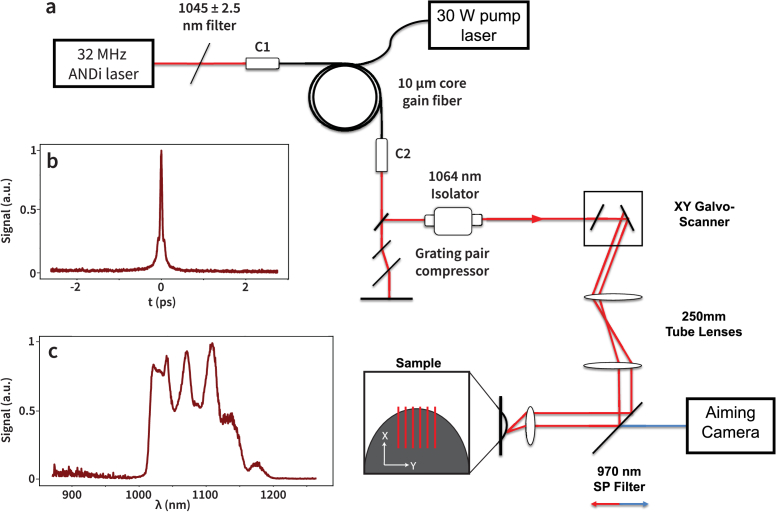
(a) Schematic of the complete optical system used for the nonlinear ablation experiments. A 32 MHz all-normal-dispersion (ANDi) laser oscillator is coupled to the input collimator (C1) and serves as the seed source for the gain-managed amplifier (GMA), which outputs pulses with sufficient energy and compressibility for ablation. A short pass (SP) filter is used to allow targeting of the ablation beam along with a camera for live-aiming. For silicon ablation, a low NA air objective was used for focusing. For cellular ablation, the beam was focused through a high-numerical-aperture (NA) microscope objective (20 × , 1.0 NA; Olympus XLUMPLFLN). (b) Autocorrelation trace showing the optimally compressed pulse duration ( ∼ 46 fs autocorrelation fit) used in ablation experiments. (c) Power spectral density of the pulse measured at the GMA output, showing a spectral bandwidth of approximately 140 nm.

*Laser system and optical configuration.* To investigate the impact of pulse duration on nonlinear ablation efficiency, we modified the femtosecond all-normal-dispersion (ANDi) fiber laser system previously developed by our group and others [33,39], and integrated an Yb-fiber-based gain-managed amplifier (GMA) (Fig. 1(a)). The seed oscillator alone produces pulses with energies in the range of 2–5 nJ and durations of 70–100 fs, which are insufficient for material ablation. However, the mode-locked output serves as an ideal seed for gain-managed nonlinear amplification.

GMA amplifiers generate high-energy pulses with bandwidth exceeding that of typical Yb-based CPA [34,35]. These amplifiers host strong nonlinear spectral broadening and a longitudinally-varying gain spectrum. Prior work shows that GMA amplifiers are insensitive to seed pulse parameters like chirp, bandwidth, center wavelength, and energy within an acceptable range [34]. Here we generate a seed pulse with 2.5 nm bandwidth at 1045 nm by spectrally filtering the pulse from the ANDi oscillator to a duration of ∼ 400 fs. The Yb-doped gain fiber has a 10 µm core diameter (Thorlabs, YB1200-10/125DC-PM), and is sufficiently long (3 m) to support a longitudinally-varying gain spectrum due to inversion depletion. Pulses generated with this amplifier have broad bandwidths (140 nm; 1020–1160 nm; Fig. 1(c)), indicating evolution in the gain-managed regime. These pulses reach energies of 90 nJ, and are compressed to < 50 fs duration using a standard Treacy compressor [40]. At the repetition rate inherent to the laser oscillator used in these experiments, 32 MHz, this yields an average laser power of ∼ 2.9 W.

The amplified pulses were directed to the sample via a pair of galvanometer scanning mirrors controlled by a custom LabVIEW interface, enabling flexible adjustment of scan speed, size, and shape. All experiments used a bidirectional raster scan pattern. The scanned beam was expanded with a telescope relay and directed orthogonally into a final focusing lens. For silicon ablation, we used a low numerical-aperture air objective (0.25 NA). For biological ablation experiments, the focusing lens was replaced with a high-numerical-aperture water immersion objective (N20X-PFH 20 Olympus XLUMPLFLN, 20 × , 1.00 NA, 2.0 mm working distance) for improved axial confinement. Table 1 summarizes all laser parameters used in the silicon and cellular ablation experiments to facilitate direct comparison of the experimental conditions.

**Table 1.** Laser parameter details for the silicon and cell ablation experiments. Estimated cell diameter = 20 µm

Laser Parameter	Silicon	Cell
Beam Waist ( $\omega_{0}$ , µm)	2.34	1.22
Rayleigh Length ( $z_{0}$ , µm)	12.9	7.1
Pulse Energy (nJ)	60	6
Scan Speed (mm/s)	200	20
Fluence (J/cm^2^)	130	320
Regional energy/scan (mJ)	10	50
[Estimated energy per cell/scan (mJ)]	-	[1.5]

*Silicon Ablation.* Ablation experiments were performed on (100)-oriented silicon wafers using a constant pulse energy of 60 nJ. Beginning with the shortest achievable pulse duration (46 fs), raster scans were conducted at a speed of ∼ 0.2 m/s across the top surface and extended beyond the edge of the wafer to generate grooves. Cross-sections of these grooves were imaged on the orthogonal face using a Zeiss LSM800 confocal microscope with a 20 × objective. The cross-sectional areas measured from these images multiplied by the laser scan speed yielded the volume ablation rate which was used as the quantitative metric of ablation efficiency. This procedure was repeated for progressively longer pulse durations while keeping pulse energy and scan parameters constant.

*Cancer cell model.* The NIH:Ovcar5 cancer cell line was obtained under a material transfer agreement from Fox Chase Cancer Center (Philadelphia, PA). Ovcar5-GFP cells stably expressing enhanced green fluorescent proteins (EGFP) were generated by lentiviral transduction following previously described protocols [41].

*2D cell culture.* Ovcar5 and Ovcar5-GFP cells were maintained in 25 ${\mathrm{cm}}^{2}$ flasks (Fisherbrand, FB012935) in RPMI 1640 medium (Gibco, 61-870-036) supplemented with 10% FBS (Sigma-Aldrich, F0926) and 1% penicillin–streptomycin (Gibco, 15140122). At 90% confluency, cells were dissociated using TrypLE Express (Gibco, 12604013), washed, and replated at 1:10 dilution. Cultures were refreshed after 30 passages from thawed vials.

*2D cell ablation.* As described above, the high-numerical-aperture (1.0 NA) water-immersion objective used for cellular ablation replaced the low NA air objective from the silicon experiments so as to provide greater confinement of the ablation beam, limiting unintended collateral damage. Due to the transmission efficiency of the objective and associated optics, the laser power at the sample plane was reduced to 185 mW. At a repetition rate of 32 MHz, this corresponds to a pulse energy of approximately 6 nJ—sufficient for cell nanosurgery ablation applications given the applied laser parameters such as laser spot size [2,18,19]. To evaluate the influence of pulse duration in a biological system, Ovcar5-GFP cells were seeded at ∼ 3 × $10^{5}$ cells/mL into glass-bottom petri dishes. On day 0, 100 µL of the cell suspension was plated and allowed to adhere for 2 hours, followed by the addition of 2 mL of fresh culture medium. After two days of growth (day 2), ablation was performed by raster scanning the laser over 100 µm × 100 µm regions at a speed of ∼ 22 mm/s, delivering approximately 2,000 pulses per optically resolved focal spot. Multiple regions (5–15 per dish) were scanned to reduce overall time outside of incubation. Propidium iodide (PI, 5 µg/mL) was added immediately following ablation and incubated for 1 hour. Fluorescence imaging was performed using a Zeiss LSM880 NLO confocal microscope with a 20 × objective. This ablation protocol was repeated across a range of pulse durations for comparative analysis.

*3D cancer model ablation.* For 3D spheroid ablation, Ovcar5-GFP cells were suspended in Matrigel, a commercial artificial cellular matrix commonly used for 3D cell culturing and imaging experiments, at ∼ 3 × $10^{5}$ cells/mL. On day 0, 10 µL Matrigel domes were plated in glass-bottom dishes. After 3 days of spheroid growth, raster scans were performed at various depths using the same average power (185 mW) and shortest pulse duration ( ∼ 50 fs) used in the 2D experiments. The scan area and speed were identical to the 2D protocol. Initial ablation was performed at the surface of each spheroid. If the spheroid was large enough to allow an additional planar scan, an additional ablation scan was performed approximately 10–20 µm deeper into the spheroid. For spheroids located beyond ∼ 350 µm depth, additional raster passes were required to achieve comparable PI uptake. This is likely due to beam attenuation and aberration in the Matrigel and embedded tumor spheroids. Matrigel has a similar refractive index to water (1.056 relative index), but otherwise unreported attenuation and absorption properties. Tumor spheroids embedded in Matrigel likely have optical properties somewhere between a nearly transparent hydrogel and tumor tissue, depending on the volume fraction of the tumor spheroids. Tissue mimicking phantoms provide a known benchmark, which exhibit the following approximate absorption and reduced scattering coefficients for wavelengths near 1080 nm: $\mu_{a}=0.15$ ${\text{cm}}^{-1}$ and $\mu_{s}^{\prime }=71$ ${\text{cm}}^{-1}$ [42,43]. Accordingly, scattering dominates attenuation at the depths examined. PI staining (5 µg/mL) and imaging were conducted as described for the 2D experiments.

## 3. Results

### 3.1. Model of pulse duration dependence of ablation efficiency in silicon

Silicon was selected as the initial test material because its optical properties, nonlinear absorption characteristics, and ablation response are well characterized in the ultrafast-laser literature. Its rigidity enables reproducible groove formation and straightforward quantitative analysis of ablation morphology, making it an ideal platform for establishing baseline trends in pulse-width–dependent ablation and optimizing laser parameters before transitioning to biological samples.

To provide a theoretical framework for interpreting our ablation efficiency experiments, we developed an analytical model based on Gaussian beam propagation, similar to that used by Wang et al. [44]. The spatial intensity distribution of a Gaussian beam in cylindrical coordinates is given by:

(1) $$I(\rho ,z)=I_{0}{\left(\frac{\omega_{0}}{\omega (z)}\right)}^{2}e^{\frac{-2\rho^{2}}{\omega^{2}(z)}}$$

where $I_{0}$ is the peak intensity at the beam center, ρ is the radial coordinate, z is the axial coordinate, ω (z) is the beam radius at axial position z, and $\omega_{0}$ is the beam waist located at the focal plane (z=0). Using the beam radius relation $\omega^{2}(z)=\omega_{0}^{2}\left(1+\frac{z^{2}}{z_{0}^{2}}\right)$ the intensity along the beam axis ( ρ = 0), can be simplified to:

(2) $$I(\rho =0,z)=I_{0}\left(\frac{z_{0}^{2}}{z^{2}+z_{0}^{2}}\right)$$

where $z_{0}$ is the Rayleigh range, the distance from the beam waist where the beam radius increases by $\sqrt{2}$ . At the beam focus (z=0), the transverse intensity distribution becomes:

(3) $$I(\rho ,z=0)=I_{0}e^{\frac{-2\rho^{2}}{\omega_{0}^{2}}}$$

To express $I_{0}$ in terms of experimentally accessible parameters, we use:

(4) $$I_{0}=P_{0}/\pi \rho^{2}$$

where $P_{0}$ is the peak power of the pulse and $\pi \rho^{2}$ is the beam’s cross-sectional area. This can then be rewritten using the beam radius relation $\omega^{2}(z)=\omega_{0}^{2}\left(1+\frac{z^{2}}{z_{0}^{2}}\right)$ . In transparent materials, ablation occurs only where the intensity exceeds a material-specific threshold, $I_{\text{th}}$ . Importantly, this assumption allows us to define ablation thresholds as:

(5) $$I(\rho =0,z_{th})=\frac{P_{0}}{\pi \omega_{0}^{2}}\frac{1}{1+z_{th}^{2}/z_{0}^{2}}=I_{th}$$

(6) $$I(\rho_{th},z=0)=\frac{P_{0}}{\pi \omega_{0}^{2}}e^{\frac{-2\rho_{th}^{2}}{\omega_{0}^{2}}}=I_{th}$$

Rearranging these equations and solving for the axial and radial distances, we define the ablation boundaries as follows:

(7) $$z_{th}(\tau )=z_{0}\sqrt{\frac{P_{0}}{\pi \omega_{0}^{2}I_{th}}-1}$$

(8) $$\rho_{th}(\tau )=\omega_{0}\sqrt{ln\frac{P_{0}}{\pi \omega_{0}^{2}I_{th}}}$$

Furthermore, we relate peak power to energy assuming a Gaussian temporal profile:

(9) $$P_{0}≃0.94\frac{E}{\tau }$$

with E as the pulse energy and τ as the pulse duration (FWHM). Substituting $E=P_{\text{avg}}/f_{\text{rep}}$ where $P_{\text{avg}}$ is the average power and ${\text{f}}_{\text{rep}}$ is the repetition rate yields expressions for the ablation threshold distances in terms of pulse duration:

(10) $$z_{th}(\tau )=z_{0}\sqrt{\frac{0.94P_{avg}}{\tau f\pi \omega_{0}^{2}I_{th}}-1}$$

(11) $$\rho_{th}(\tau )=\omega_{0}\sqrt{ln\frac{0.94P_{avg}}{\tau f\pi \omega_{0}^{2}I_{th}}}$$

To evaluate the model, we first determine three key parameters: $\omega_{0}$ , $z_{0}$ , and $I_{\text{th}}$ . The ablation threshold for silicon has been empirically reported in literature. Based on the close match between our system and that of Taylor et al. [45], we adopt $I_{\text{th}}$ = 0.43 ${\mathrm{J/cm}}^{2}$ for 300 fs pulses at ∼ 1030 nm. To determine $\omega_{0}$ and $z_{0}$ , we measured the point spread function (PSF) using two-photon excitation of 0.2 µm fluorescent beads, employing the same optical system used for ablation. From PSF image analysis (see Supplement 1), we obtained $\omega_{0}$ = 2.34 µm and $z_{0}$ = 12.9 µm. Using these parameters in the threshold equations, we calculated the theoretical axial and transverse ablation radii as a function of pulse duration. Due to the available laser scanning speed being sufficiently slow with respect to the laser repetition rate and the difficulties with directly measuring the depth of the ablation grooves with traditional imaging methods, the cross-sectional area of the grooves was chosen to be directly measured. Because the experiments were conducted at the sample surface rather than fully embedded in the material, we applied a correction factor of $\frac{1}{2}$ to the calculated ablation area to account for only half of the elliptical focus intersecting the target. This cross-sectional area was finally multiplied by the laser scan speed (v = 200 mm/s) to determine the volumetric ablation rate, V, which was used to model the measured ablation rates in silicon:

(12) $$V(\tau )=v\times \frac{\pi \rho_{th}z_{th}}{2}$$

**Fig. 2.**
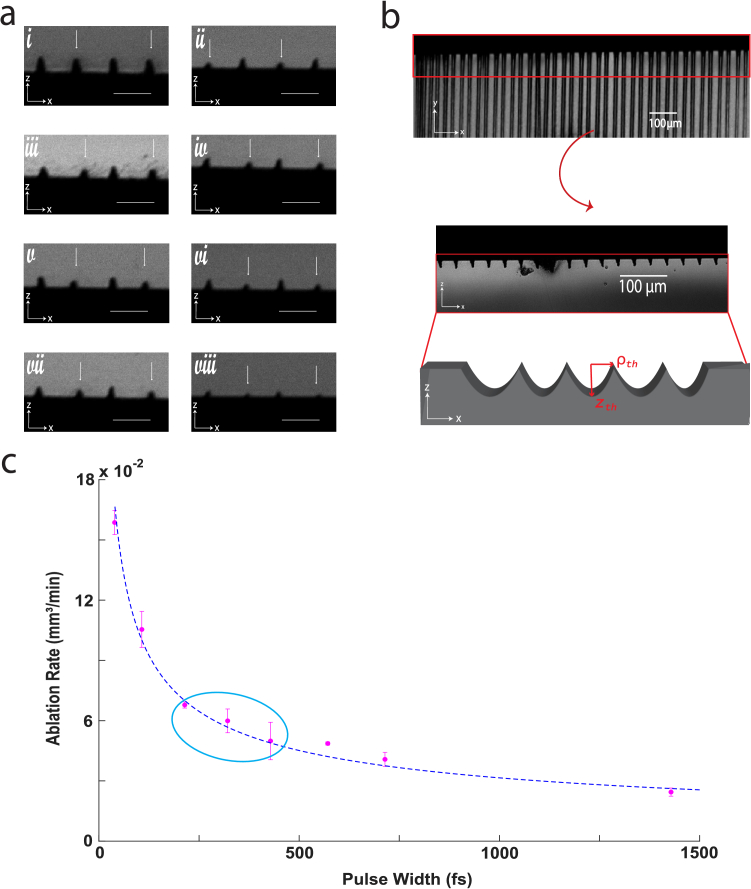
(a) Cross-sectional areas depicting the varying morphologies of ablation grooves generated by ablation using pulse widths of approximately 46, 110, 214, 319, 431, 571, 714, and 1431 fs (i-viii). Arrows denote the grooves generated at 0.2 m/s scan speed. Scale bars are 20 µm. (b) One technical replicate acquired at 46 fs showing the cross-sectional areas that were measured. (c) Silicon ablation experiments reveal a clear increase in material ablation with decreasing laser pulse duration. The ablation laser delivered pulses at 32 MHz with a constant energy of 60 nJ per pulse. The blue ellipse highlights the typical pulse durations used in commercially available systems with similar pulse energies. Cross-sectional areas for each groove were measured as shown in panel (b) and the mean ablation rate for each pulse duration is reported here. Results are mean ± s.e.m. (*n*=4 technical replicates, $\chi_{red}^{2}=10.4)$ . Given the relative simplicity of this model and the relatively large reduced chi-squared value, the model may underfit the data. With constant scan speed and stable beam shape, this ablation rate serves as a reliable metric of ablation efficiency, demonstrating the advantages of ultrashort pulses in the mid-repetition-rate regime.

As shown in Fig. 2(c), the theoretical model demonstrates reasonable agreement with our experimental measurements of ablation area as a function of pulse duration. This alignment supports the conclusion that ablation efficiency can be enhanced by reducing pulse duration, without increasing average laser power. The relatively high chi-squared value for the model fit likely reflects an underfitting of the data due to simplifying assumptions. In particular, the model assumes that the cross-sectional shape of the ablation grooves remains consistent across pulse durations. However, at the shortest pulse durations, the grooves become more sharply pointed and triangular, in contrast to the smoother, hemispherical profiles observed at longer durations (Fig. 2(a)).

Although our model correctly predicts that groove depth should increase more rapidly than width as pulse duration decreases, the experimentally observed morphological variation was more pronounced than anticipated. This behavior reflects the combined influence of ultrashort pulse durations, the near-infrared wavelength at which silicon exhibits minimal linear absorption, and the high pulse count delivered at 32 MHz. In this regime, shorter pulse durations substantially increase peak intensities, enhancing multiphoton absorption and lowering the effective ablation threshold, consistent with prior studies across various materials [28,29,31,32,46–48]. Because each focal position receives many pulses (>300) at the scan speed used here, incubation effects become a dominant contributor to material modification and removal [14,15,25,30,49], amplifying the efficiency gains associated with shorter pulse durations. In contrast, longer pulses exhibit reduced nonlinear absorption and weaker incubation effects, leading to higher effective ablation thresholds and shallower penetration depths, which is consistent with the shallow grooves observed experimentally.

The narrower trench profiles produced at the shortest pulse durations likely arise from limitations of the scanning hardware rather than intrinsic material behavior. The relatively slow galvo mirror speed results in prolonged exposure of the recently ablated region to the trailing portion of the pulse train, causing localized heating and less efficient material ejection [27,50]. While a comprehensive mechanistic model of pulse-duration-dependent groove morphology lies beyond the scope of this work, the observed trends align with prior theoretical and experimental studies and support the conclusion that shorter pulses improve ablation efficiency without necessitating higher average powers. These results provide a controlled and interpretable testbed for defining pulse-duration–dependent ablation behavior within this mid-repetition rate regime. Having established these trends in a well-characterized material, we next extend our investigation to biological systems to assess how pulse duration influences microsurgical ablation in a cellular environment.

### 3.2. Cellular nonlinear laser ablation

From the silicon ablation studies, we established the pulse-duration–dependent scaling of ablation efficiency and identified the pulse energies and scan speeds that produced reliable material removal under our laser conditions. These trends translate to biological systems by providing a basis for selecting scan speeds and energy densities that remain effective for cellular ablation while avoiding excessive collateral damage. With this experimentally grounded baseline defined in a well-characterized material, we next extended the approach to cultured cells to evaluate how pulse duration influences microsurgical ablation in a biologically relevant context.

For biological validation, we applied these laser conditions to cancer cell cultures. In vitro cancer cells were chosen because they are robust, scalable, and amenable to live/dead fluorescence assays, allowing high-throughput assessment of ablation efficacy and cellular damage. We used both 2D monolayers—representing surface-accessible tissue geometries relevant to many surgical and dermatologic targets—and 3D spheroid cultures embedded in Matrigel, which approximate key features of tumor nodules and extracellular matrix composition. These 3D constructs provide a more physiologically relevant environment in which to assess depth targeting, energy confinement, and multicellular-scale ablation behavior.

At this resolution, we deliberately forgo the single-cell and subcellular precision demonstrated in femtosecond nanosurgery [1,2,6,9,22] in order to achieve faster, region-scale ablation suitable for microsurgical applications. This trade-off enables us to scan larger areas efficiently while still preserving a moderately precise, spatially confined ablation effect. As a result, the methods used here offer a step towards substantially improved localization and reduced collateral damage compared to macroscopic, thermally driven clinical ablation modalities such as laser interstitial thermal therapy (LITT) and radiofrequency ablation (RFA) [51–54].

As described in the methods section, the ablation laser was raster scanned across the plasma membranes of Ovcar5-GFP cells within the field of view. During these experiments, we observed a marked decrease in ablation efficiency with increasing pulse duration. To obtain comparable membrane damage across different pulse durations, the number of raster scans was adjusted accordingly to produce similar final ablation results as determined through experimentation.

In silicon, a single scan cannot penetrate the full wafer thickness. In contrast, the determined rayleigh length for the objective used in these cell ablation experiments ( ∼ 7.1 µm) is significantly larger than the typical thickness of a cell membrane ( ∼ 10 nm), allowing complete membrane perforation with a single scan. While direct measurement of ablated membrane area would require higher resolution imaging (e.g., cryoelectron microscopy), the extent of irreversible membrane damage, as indicated by propidium iodide uptake, provides a practical and biologically relevant measure of ablation efficiency.

**Fig. 3.**
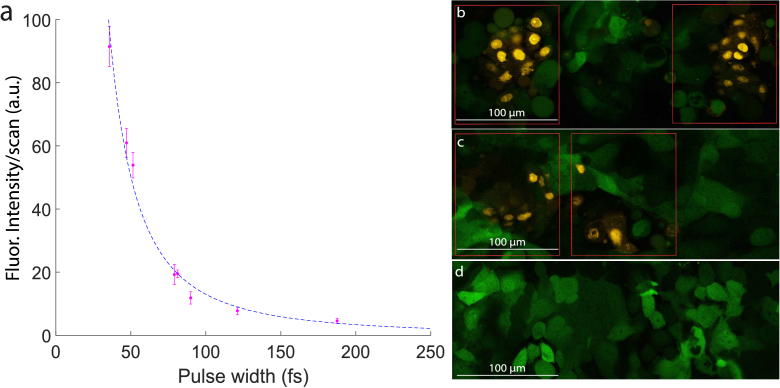
The dependence of laser ablation efficiency on pulse duration was evaluated in Ovcar5-GFP cell cultures using propidium iodide (PI) fluorescence as a marker of membrane disruption. All ablation experiments were performed using laser pulses delivered at 32 MHz with a constant pulse energy of 6 nJ . (a) The mean PI fluorescence intensity per ablation laser raster scan was quantified by fluorescence microscopy and plotted as a function of pulse duration ( τ ). A $\tau^{-2}$ trendline is overlaid to illustrate the approximate relationship between pulse duration and ablation efficiency. For each of these replicates the estimated energy deposited within each cell is 1.5 mJ per raster scan. Results are mean ± s.e.m. (*n*=3 biological replicates, $\chi_{\text{red}}^{2}=1.325$ ). (b–d) Representative fluorescence images acquired 1 hour post-ablation. Red rectangles in (b) and (c) indicate the ∼ 10,000 ${\mathrm{µm}}^{2}$ ablation region used for intensity quantification. (b) Exemplary image for a 50 fs pulse duration. (c) Representative image for a 170 fs pulse duration. (d) Negative control image with no ablation.

After laser exposure, propidium iodide (PI) fluorescence imaging was used to assess the extent of membrane compromise. Propidium iodide is cell impermeant and largely non-fluorescent while free in suspension and exhibits strong fluorescence once uptaken by cells whose membranes are sufficiently disrupted to allow the PI stain to flow in and intercalate DNA, allowing us to easily distinguish between live and dead cells. The average PI fluorescence intensity was quantified within the ∼ 10,000 ${\mathrm{µm}}^{2}$ ablation scan region (outlined in red in Figs. 3(b) and 3(c)). To account for the varying number of raster scans used at each pulse duration, the measured fluorescence intensities were normalized by the number of scans, yielding a metric for ablation efficiency per scan.

Directly measuring the pulse duration at the focal plane of the high-NA microscope objective proved impractical due to significant group velocity dispersion (GVD) in the optical system, which introduced background in our autocorrelation setup and prevented reliable characterization at the focus. Instead, we empirically optimized pulse compression by maximizing two-photon excitation of sulforhodamine 101 (SR101), a dye well matched to our laser’s bandwidth. We then used this pulse condition as a reference point and estimated longer pulse durations via temporal Strehl ratio calculations, as shown in Fig. 3.

While a complete theoretical model of the biological mechanisms involved in cell membrane ablation is beyond the scope of this study, the empirical dependence of PI fluorescence on pulse duration is clear. The data exhibit an approximately inverse-square relationship between fluorescence intensity and pulse duration, contrasting with the silicon ablation experiments, where ablation was observed to be more directly influenced by the beam’s spatial geometry as it compares to the material thickness.

The observed inverse-square dependence on pulse duration agrees with that seen in multi-photon imaging processes, which exhibit a $1/\tau^{\left(n-1\right)}$ , relation where n is the number of photons required for the process [33,38,55,56]. The nonlinear dependence on pulse duration observed in these experiments ( $∼\tau^{-2}$ ) supports that this ablation process is likely a multiphoton phenomenon. Furthermore, because the average power was held constant throughout our experiments, the peak power in these experiments is therefore inversely proportional to pulse duration. Thus the ablation efficiency ( $A_{\text{eff}}$ ) proportionality can be written:

(13) $$A_{eff}\propto \tau^{-2}\;\text{or}\;A_{eff}\propto P_{0}^{2}$$

Provided this proportionality and the assumption that this ablation is a multiphoton process, we can conclude that ablation efficiency is dominated by peak pulse power rather than average power in the regime of moderate repetition rate and ultrathin biological targets.

**Fig. 4.**
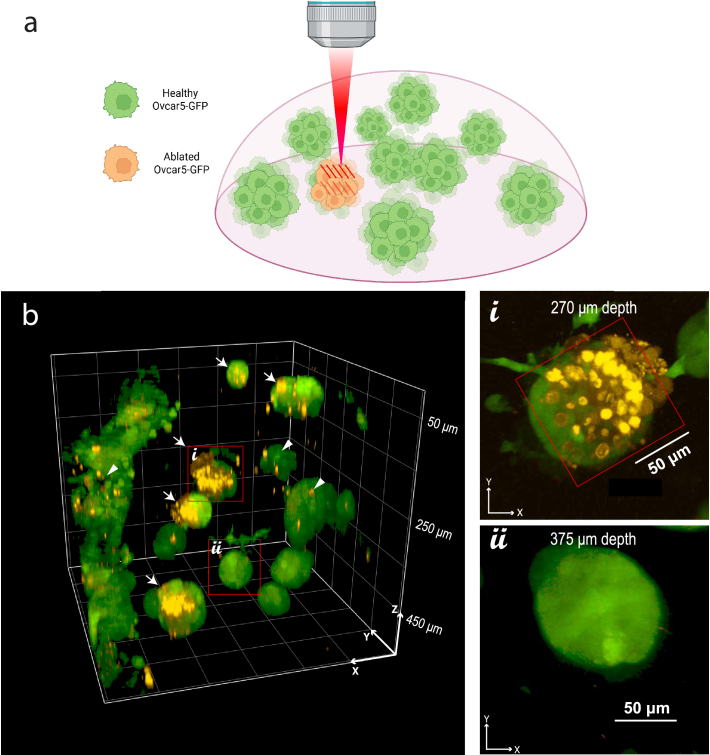
(a) Schematic representation of the 3D Ovcar5-GFP ablation experimental process. (b) Fluorescence imaging of 3D Ovcar5-GFP cell cultures demonstrates the depth-resolved localized ablation enabled by ultrashort laser pulses. All ablation was performed using pulses delivered at 32 MHz with a surface pulse energy of 6 nJ (n=4 replicates, 2 biological replicates). Ablation laser scan number was increased to maintain effective ablation at depth. Arrows pointing to the right indicate cells that were successfully ablated, as evidenced by PI uptake. Arrowheads pointing to the left identify PI-positive cells that were not targeted by the laser and represent basal levels of cell death in the model. Spheroids shown exhibited a mean diameter of 82.7 ± 4.4 µm (n=16, mean ± s.e.m.) (i) Zoomed-in maximum intensity projection of an ablated spheroid ∼ 270 µm deep, showing PI-positive cells extending to a depth of approximately 75 µm within an individual tumor nodule. The red square denotes the 100 µm × 100 µm ablation region. (ii) Zoomed-in projection of a neighboring non-ablated control spheroid showing no evidence of laser-induced damage.

A practical demonstration of this cellular ablation technique is shown in Fig. 4, where we performed targeted ablation within 3D Ovcar5-GFP spheroids embedded in Matrigel, a matrix that mimics extracellular tissue architecture. Using 46 fs pulses (the shortest and most effective pulse duration achieved in this study, Fig. 3), we ablated spheroids at varying spheroid depths, performing planar raster scans at one or two planes within the spheroid depending on the spheroid size, while minimizing off-target effects on neighboring structures.

In an effort to validate the depth resolution of this ablation process, z-axis intensity profiles were generated for three of the spheroids shown in Fig. 4 and fit to the square of the axial intensity profile of a gaussian beam as determined by the proportionality in Eq. (13).

(14) $$I{\left(z\right)}^{2}=I_{0}^{2}{\left(\sqrt{1+{\left(\frac{z}{z_{eff}}\right)}^{2}}\right)}^{-4}$$

where z is the axial location of the beam focus and $z_{\text{eff}}$ is the effective axial ablation range.

**Fig. 5.**
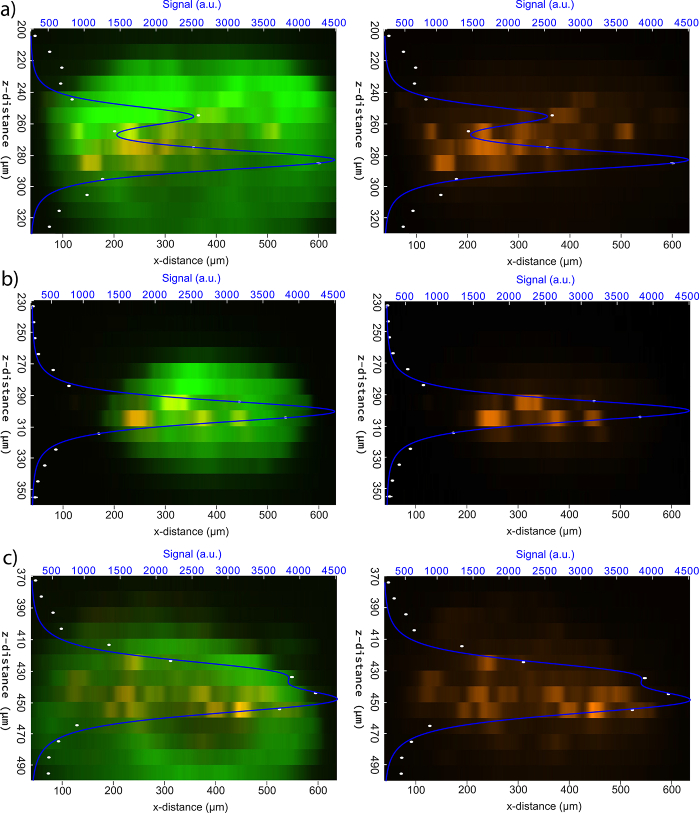
Combined GFP + PI dye intensity profiles (left column) and the isolated PI dye intensity profiles (right column) along the depth of the ablated spheroid for a three of the spheroids seen in Fig. 4. The maximum one percent of PI dye intensities across each z-slice (slice width = 9.18 µm) were plotted and fit to the axial gaussian beam intensity profile described in Eq. (14). a) Z-profile of the GFP+PI intensity and isolated PI intensity along with the associated axial intensity fit for the spheroid starting at a depth of 200 µm. This spheroid was subject to two planar ablation scans. b) Z-profile of the GFP+PI intensity and isolated PI intensity along with the associated axial intensity fit for the spheroid starting at a depth of 230 µm. This spheroid was subject to a single planar ablation scan. c) Z-profile of the GFP+PI intensity and isolated PI intensity along with the associated axial intensity fit for the spheroid starting at a depth of 370 µm. This spheroid was subject to two planar ablation scans.

The spheroids depicted in Fig. 5(a) and (c) were ablated at two separate planes and were therefore subject to a fit that is a modified 2-plane version of Eq. (14) where an additional term is used to model an additional axial intensity centered at a separate axial location. These fits provide estimates of the effective axial range and 95% confidence error for the ablation planes in each of these spheroids: a) z = 11.41 ± 2.48 µm; b) z = 12.02 ± 0.92 µm; c) z = 14.17 ± 4.59 µm. These values are reasonably similar to the Rayleigh length of the objective used for these ablation experiments, 7.1 µm. The disparity between these calculated values and the measured Rayleigh length likely stems from the distortion to the beam after propagation through the scattering Matrigel dome and the additional depth that an ablated spheroid contributes to this measurement. The disrupted beam quality required the use of additional raster scans (12-22 scans) beyond that used in the 2D ablation experiments (2 scans), possibly leading to localized heating and collateral damage extending beyond the axial location of the beam focus. Notably the axial control seen in the spheroid in Fig. 5(c)) is comparatively worse than that seen in the other two spheroids. This can be attributed to the further disruption to the beam quality at this depth, requiring an increased raster scan number (22 raster scans) to achieve ablation, likely generating further excess heat and in turn collateral damage. While these preliminary 3D ablation experiments were limited in scope, they demonstrate the potential for localized microsurgery in more biologically relevant models, paving the way for future studies into the broader biological consequences of ultrafast laser ablation.

## 4. Conclusion

This study demonstrates that reducing pulse duration in the mid-repetition-rate regime substantially enhances nonlinear laser ablation efficiency in both bulk materials and biological systems. Using a custom-built femtosecond fiber laser system, featuring gain-managed amplification and capable of delivering 90 nJ, 46 fs pulses at 32 MHz, we have accessed an underexplored region of ablation parameter space. In silicon, the efficiency trends align well with theoretical predictions based on spatial beam intensity. In contrast, cellular membrane ablation appears more strongly governed by pulse peak power, implicating pulse duration as a critical factor in achieving membrane permeabilization at low pulse energies.

These findings suggest that shortening pulse duration is not merely a matter of marginal optimization, but a fundamental mechanism for increasing ablation efficiency—especially when average laser power must be constrained to avoid thermal damage [53,57–59]. In biological media, this strategy also reduces cavitation bubble formation [17], a known contributor to off-target effects, and allows ablation at depth within 3D tumor models without damaging adjacent structures. Such minimally disruptive, targeted microsurgery could inform future applications in image-guided oncology or regenerative medicine.

Importantly, this work supports a broader shift toward ablation cooling strategies, such as ultrafast pulse bursts (GHz), where ultrashort pulses can further confine energy deposition spatially and temporally to achieve the fastest ablation speeds with minimal collateral damage [26,60]. While practical limits in pulse compression and system complexity remain, the data presented here underscore the potential of sub-50-fs pulses to improve both the speed and selectivity of laser microsurgery. Continued exploration of this regime may ultimately help bridge the gap between laboratory ablation tools and clinically viable systems.

## Supplemental information

Supplement 1 Supplement 1 https://doi.org/10.6084/m9.figshare.30797849

## Data Availability

The raw data underlying the results presented in this paper are not publicly available at this time but may be obtained from the authors upon reasonable request.

## Supplemental document

See Supplement 1 for supporting content.
