# Supplementary material for: Sub-50-femtosecond gain-managed amplified pulses enhance nonlinear ablation efficiency
Source: Biomed Opt Express. 2025 Dec 10;17(1):202–16. doi: 10.1364/BOE.582102 (PMC12795435; doi:10.1364/BOE.582102)
Supplement: Supplementary file 1 [file boe-17-1-202-s001.pdf]

Supplemental Document

Biomedical Optics EXPRESS

# Sub-50-femtosecond gain-managed amplified pulses enhance nonlinear ablation efficiency: supplement

LIAM J. PRICE,[1,2] KAI ZHANG,[1,2] NICHOLAS J. OTERO,[1,2] PAUL REPGEN,[3,4] PABLO A. VALDES,[5] FRANK WISE,[6] FATIH ÖMER ILDAY,[3,4] AND BRYAN Q. SPRING[1,2,7,*]

[1]*Translational Biophotonics Cluster, Northeastern University, Boston, Massachusetts 02115, USA*
[2]*Department of Physics, Northeastern University, Boston, Massachusetts 02115, USA*
[3]*Department of Physics and Astronomy, Ruhr University Bochum, 44801 Bochum Süd, Germany*
[4]*Department of Electrical Engineering and Information Technology, Ruhr University Bochum, Bochum Süd 44801, Germany*
[5]*Department of Neurosurgery, University of Texas Medical Branch, Galveston, Texas 77555, USA*
[6]*Department of Applied and Engineering Physics, Cornell University, Ithaca, New York 14850, USA*
[7]*Department of Bioengineering, Northeastern University, Boston, Massachusetts 02115, USA*
[*]*b.spring@northeastern.edu*

This supplement published with Optica Publishing Group on 10 December 2025 by The Authors under the terms of the Creative Commons Attribution 4.0 License in the format provided by the authors and unedited. Further distribution of this work must maintain attribution to the author(s) and the published article's title, journal citation, and DOI.

Supplement DOI: https://doi.org/10.6084/m9.figshare.30797849

Parent Article DOI: https://doi.org/10.1364/BOE.582102

# Sub-50-femtosecond gain-managed amplified pulses enhance nonlinear ablation efficiency: supplemental document

PSF analysis was performed for both ablation objectives described in the main manuscript. 0.2 µm diameter fluorescent beads were imaged with both objectives using the galvo-scanning mirrors to scan the beam for imaging. The process for this PSF analysis follows previous work [1] that serves as a general example of PSF analysis. The intensity profiles of the fluorescent beads in the x and y planes were fit with a gaussian profile to determine the PSFs in each direction. Multiple frames across the depth of the fluorescent beads were recorded and used to fit the intensity profile along the z-direction to determine the z PSF. Fits of the x, y, and z PSFs were used to estimate the beam waist and associated Rayleigh lengths for each objective. Fluorescent images of the beads and associated PSF fits can be seen in Fig. S1. Because the ablation scan direction was along the x-direction, the width of this dimension did not contribute to the width of the created ablation groove. As a result, the reported beam waists are determined from the y PSF fits. The reported Rayleigh lengths are similarly determined from the z PSF fits. The waist values in the transverse directions along with the axial Rayleigh lengths are provided in Table S1.

| | 0.25 NA air objective | 20×, 1.0 NA; Olympus XLUMPLFLN |
|---|---|---|
| $\omega_x$ (µm) | 2.07 | 1.25 |
| $\omega_y$ (µm) | 2.34 | 1.22 |
| $z_R$ (µm) | 12.91 | 7.1 |

**Table S1.** Beam waist and Rayleigh lengths calculated for both ablation objectives.

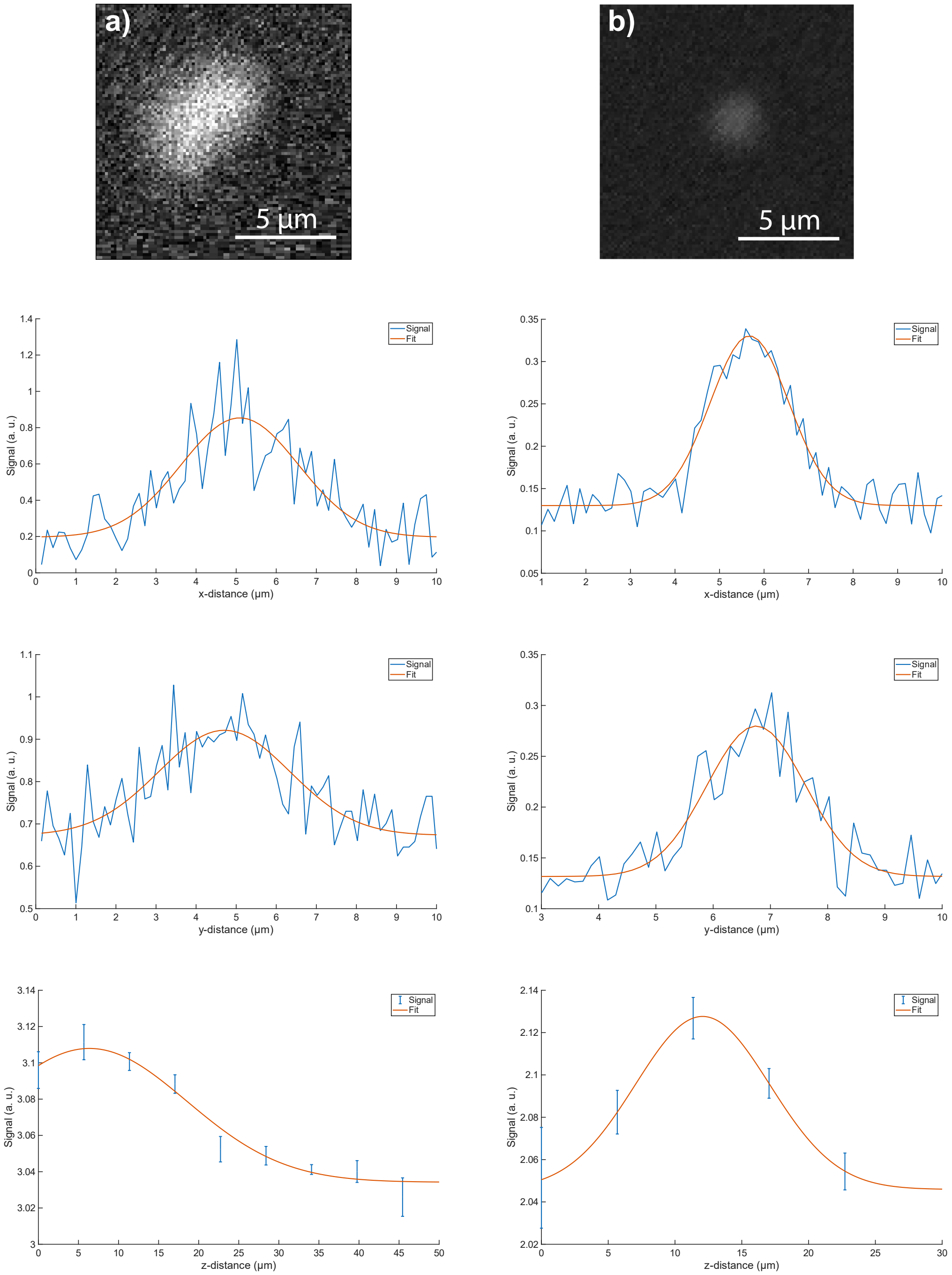


**Fig. S1.** PSF analysis of the two objectives used in ablation experiments. a) 0.2 µm diameter fluorescent bead and the associated x, y, and z PSF fits for the 0.25 NA objective used in the silicon ablation experiments. a) 0.2 µm diameter fluorescent bead and the associated x, y, and z PSF fits for the 1.0 NA objective used in the cell ablation experiments.

## REFERENCES

1. K. Zhang, G. Ducourthial, T. Sharan, *et al.*, “Video-rate two-photon microendoscopy using second harmonic resonance fiber scanning,” Biomed. Opt. Express **15**, 6324–6339 (2024).
